# Influenza

**Published:** 1907-04

**Authors:** 


### INFLUENZA.

---

The Practitioner for January, 1907, entitled Special Influenza Number, has come to hand. It has not been our good fortune for some time to peruse such an interesting and instructive series of articles. The list of authors is sufficient guarantee that every article will amply repay careful study.

It is not possible in a review to cover all the ground taken up in this number of the Practitioner. The more salient features, as we see them, will be briefly considered.

HISTORY.—The present epidemic of influenza seems to be one of the "flarings" that has followed the great pandemic of 1889-1890. The fact that from 1848 to 1889 there was practically no influenza may be accounted for by the very limited means of transportation during that period. The increase of population and the rapid means of travel favor the wide-spread dissemination of such a disease as influenza.

ETIOLOGY.—The Bacillus influenza (Pfeiffer) is now considered to be the cause of the disease. It can be found in pure culture in the nasal and bronchial secretions of patients. Probably it is by means of these secretions that the organism is conveyed from one person to another. At any rate there is no doubt of the highly infectious nature of the malady. It seems to have a predilection for the weak spot in an individual and attacks him there most vigorously. One possible source of influenza is, perhaps, sleeping berths or sleeping-compartments in trains [another, we believe, is the absolutely non-ventilated, filthy street car, heated usually to fever temperature]. There is no immunity. Persons who have had the disease seem more apt to acquire it again when exposed.

MORTALITY.—Influenza itself is not a very fatal disease, especially when the enormous number of people attacked is taken into consideration. The heart and then the lungs are the seat of fatal complications. "Influenza poisons the heart." All are familiar with the sudden collapse that occurs in an otherwise mild attack. In old persons this is especially fatal.

SYMPTOMATOLOGY.—A disease so protean in its manifestations as influenza does not lend itself easily to a concise description of symptoms. Several main types stand out most prominently.

"1. The neurotic, neuralgic, or rheumatoid type.

"2. The cardiopulmonary type, in which the ebbing of the strength in elderly people is sometimes awful, often absolutely beyond control.

"3. The gastric or gastrointestinal type, in which anorexia is present, sometimes to the extent of a loathing for all food.

"4. The febrile type, which prevails especially among children. The febrile movement usually lasts two or three days, and is succeeded by marked subnormal temperatures, so constantly present as to become an important diagnostic sign."

The following description of the chief symptoms is so choice that it is quoted in full. "A person apparently in perfect health is suddenly overcome by a feeling of general discomfort and profound depression. He feels chilly, or is shaken with a rigor worthy of an ague fit. His head aches. There are pains in his eyeballs, or 'behind the eyes,' and the eyeballs are exquisitely tender on pressure. Soon rheumatoid pains rack his body. They are particularly severe in the nape of the neck, small of the back, in the knees, and along the margins of the ribs. The patient is often sleepless, sometimes delirious. He may temporarily suffer loss of the special senses of taste, smell, and sometimes hearing. His eyes smart, tears overflow, he shrinks from the light (photophobia). Occasionally there is inense earache (otalgia). Strength ebbs away far more quickly that even in typhus, so that, after a few hours, he must take to his bed. Nor do the digestive and respiratory system escape. The tongue becomes thickly coated with a creamy or blanketlike fur. There is a bad taste in the mouth, the breath is heavy or even foetid. There is a complete loss of appetite, amounting to a loathing against food. Nausea, and perhaps vomiting, add to the sufferer's distress. The bowels are constipated, but occasionally diarrhœa sets in, as in an attack of cholerine. In another group of cases, the brunt of the poison appears to fall on the respiratory tract—cough, expectoration, a sense of oppression on the chest, a tightness in the breathing, being common symptoms. . . . . If the patient is neurotic, nervous and neuralgic symptoms are likely to develop."

COMPLICATIONS.—These are numerous and most important. In the respiratory tract are several interesting changes. The larynx is nearly always affected but the affection of the trachea is a more distinctive feature of the disease. There is diffuse injection of the mucous membrane, and a dry cough which may be so paroxysmal as to remind one of pertussis. The cough may persist long after the acute stage has passed. In the lungs, broncho-pneumonia is the common complication. It occurs in numerous scattered areas of lobular consolidation. These may fuse together and later small abscesses may form. Consolidation of the lobar type is also seen. This usually occurs along with the pneumococcus. Streptococci are frequently found. In bronchopneumonia there is seldom an initial rigor. There is marked dyspnoea, rapid pulse, and fever of moderate grade which falls by lysis after a few days. A curious variety of "sticky" rales is characteristic of this condition.

Pleurisy occurs in from 12 to 33 per cent. of cases. A form of primary influenzal pleurisy has been described. Dry, serofibrinous, and purulent forms (empyema) occur.

The sinuses connected with the nasal cavities are very frequently severity. Optic neuritis, nerve palsies, convulsions, encephalitis, etc., melancholia, suicidal tendencies, acute manias, etc., are among the number. A very common sequela is loss of memory and an intense inertia of mind and body, which may last for weeks after the acute illness and may quite suddenly disappear. As a rule the prognosis in the influenza psychoses is good.

Ocular and Aural complications, excepting otitis media, are not of much importance. The inflammation in the middle ear arises from extension along the Eustachian tube.

PROPHYLAXIS.—Attention is called to the value of quinine as a prophylactic in epidemics of influenza. It is given in doses of two grains every morning. Where the disease has been epidemic near some institution it has been successfully kept out of the institution by isolation of the inmates.

PROGNOSIS.—This is good. The death rate from influenza itself is hardly more than 2 per cent. In 1891 when there was the highest death rate in England, there were 574 deaths in every million of the population. Influenza is responsible for a large indirect increase in the death rate. Respiratory diseases particularly show a high mortality during the periods when influenza is epidemic.

TREATMENT.—Most important of all measures is absolute rest in bed during the attack and for some time after all active symptoms have subsided. This is particularly important when there have been respiratory complications. The methods of treatment common to all beginning fevers are indicated. All the authors recommend quinine in some form. One drachm of ammoniated quinine with two drachms of liquor ammoniae acetatis may be given every hour for three hours then every four hours. Symptoms are to be treated as they arise. It must be remembered that no depressing coal-tar product should be given unless the effect is most carefully watched.—L. M. WARFIELD, in St. Louis Medical Review, February 16th, 1907.

### VERTIGO: HERAPEUICS.

By W. C. Abbott. M. D., Chicago, Ill.

Cerebral anemia is most quickly relieved by glonia, gr. 1-25o every five minutes till the face flushes or the head feels full.

The effect of glonoin is soon past but may be prolonged by giving at the same time atropine gr. 1-500 every half hour till the mouth begins to feel dry.

Cerebral anemia demands brucine or strychnine to sustain va scular tensions dose of either being gr. 1-134 repeated at short intervals till the desirable tonicity is attained.

The anemia is relieved by iron arsenate gr. 1-67 every two hours, or gr. 1-6 an hour before each meal, adding nuclein to retain the metal in the body.

Strychnine, quinine and iron arsenates with nuclein solution, make a very useful combination for those who must employ multiple therapeutics.

The vertigo of atheroma or of syphilis is relieved by corrosive sublimate or mercury biniodide, of either gr. 1-67 four to seven times each day.

Plethoric forms call for quick and powerful purgation—elaterin in full doses being the best when haste is imperative, as when apoplexy threatens.

In plethora not so acute give colchicine gr. 1-134 before meals and at bedtime, just enough to act perceptibly on the bowels or cause a little nausea.

Paroxysmal vertigo is relieved by solanine, gr. 1-12 every hour for three to ten doses, best taken before each meal and at bedtime.

Vertigo from defective renal excretion may be temporarily relieved by a full hypo of pilocarpine, a quick purge, or a saline enema; but demands investigation.

Vertigo from full colon and rectum may be relieved by a full purgative of jalapin, and a small enema of saturated salt solution.

Vertigo from the stomach is amenable to emetine, gr. 1-67 every half hour or 1-6 to 1 grain at bedtime, not as an emetic but retained.

A grain of emetine given as an emetic, in warm water, will often reveal the cause of the malady and effect its cure at the same time.

Vertigo on rising to urinate is perilous and may result in death; strengthen the va scular tone by an evening dose of digitalin or its congeners---but give dose enough.

More cases of vertigo come from gastric disorders than from all other causes combined.

Elimination, disinfection, digestion and general tonics essential to cure.

